# Detection of Organohalide-Respiring Enzyme Biomarkers at a Bioaugmented TCE-Contaminated Field Site

**DOI:** 10.3389/fmicb.2019.01433

**Published:** 2019-06-27

**Authors:** Gretchen L. W. Heavner, Cresten B. Mansfeldt, Michael J. Wilkins, Carrie D. Nicora, Garrett E. Debs, Elizabeth A. Edwards, Ruth E. Richardson

**Affiliations:** ^1^School of Civil and Environmental Engineering, Cornell University, Ithaca, NY, United States; ^2^Biological Sciences Division, Pacific Northwest National Laboratory, Richland, WA, United States; ^3^Department of Chemical Engineering and Applied Chemistry, University of Toronto, Toronto, ON, Canada

**Keywords:** reductive dehalogenase, organohalide respiration, *Dehalococcoides*, trichloroethene, biomarkers, *Geobacter*, proteomics, KB-1

## Abstract

RNA-based biomarkers have been successfully detected at field sites undergoing *in situ* bioremediation, but the detection of expressed enzymes is a more direct way to prove activity for a particular biocatalytic process of interest since they provide evidence of potential *in situ* activity rather than simply confirming presence and abundance of genes in a given population by measurement of DNA copies using qPCR. Here we successfully applied shotgun proteomics to field samples from a trichloroethene (TCE)-contaminated industrial site in southern Ontario, Canada that had been bio-augmented with the commercially available KB-1^TM^ microbial culture. The KB-1^TM^ culture contains multiple strains of *Dehalococcoides mccartyi* (*D. mccartyi*) as well as an organohalide respiring *Geobacter* species. The relative abundances of specific enzymatic proteins were subsequently compared to corresponding qPCR-derived levels of DNA and RNA biomarkers in the same samples. Samples were obtained from two wells with high hydraulic connectivity to the KB-1^TM^-bioaugemented enhanced *in situ* bioremediation system, and two control wells that showed evidence of low levels of native organohalide respiring bacteria (OHRB), *Dehalococcoides* and *Geobacter*. Enzymes involved in organohalide respiration were detected in the metaproteomes of all four field samples, as were chaperonins of *D. mccartyi*, chemotaxis proteins, and ATPases. The most highly expressed RDase in the bioaugmentation culture (VcrA) was the most highly detected enzyme overall in the bioaugmented groundwater samples. In one background groundwater well, we found high expression of the *Geobacter pceA* RDase. The DNA and RNA biomarkers detected using qPCR-based assays were a set of orthologs of *Dehalococcoides* reductive dehalogenases (VcrA, TceA, BvcA, dehalogenase “DET1545”), and the Ni-Fe uptake hydrogenase, HupL. Within a sample, RNA levels for key enzymes correlated with relative protein abundance. These results indicate that laboratory observations of TCE-bioremediation biomarker protein expression are recapitulated in field environmental systems and that both RNA and protein biomarker monitoring hold promise for activity monitoring of *in situ* populations of OHRB.

## Introduction

Chlorinated solvents are a key contaminant in shallow groundwater and soils across much of the US (Lash et al., 2000). The ability of certain microbial taxa to perform dechlorination reactions has offered an opportunity to leverage microbial metabolism for the effective remediation of such sites. However, assessing the activity, and thus efficacy, of the microorganisms that catalyze the key dechlorination reactions in complex heterogeneous media is an ongoing challenge.

Biomarkers are biomolecules (e.g., DNA, RNA or proteins) that correspond to a specific microbial population, process, or state. As early as 2001, researchers observed a strong correlation between the presence of the *Dehalococcoides mccartyi* (*D. mccartyi*) 16S rRNA gene at a site undergoing bioremediation and the reductive dechlorination of chlorinated ethenes to ethene (Hendrickson et al., 2002). 16S rRNA gene sequences are highly conserved among strains of *D. mccartyi*, forming a distinct phylogenetic group of obligate organohalide respiring organisms (Löffler et al., 2013). *D. mccartyi* strains are subdivided into three phylogenetic subgroups: Cornell (e.g., strain 195), Victoria (e.g., strain VS), and Pinellas (e.g., strains CBDB1, BAV1, GT, and FL2), based on sequence signatures in hypervariable regions 2 and 6 of the 16S rRNA gene (Hendrickson et al., 2002; Hug et al., 2011; Löffler et al., 2013). However, the presence of the *D. mccartyi* 16S rRNA gene alone does not necessarily indicate that a specific dechlorination processes will occur. The capability of using specific chloroethene electron acceptors differs among strains with nearly identical 16S rRNA gene sequences (phylogeny does not predict physiology within *D. mccartyi*). This complicates the use of the 16S rRNA gene alone to document that complete dechlorination of TCE is occurring at a certain site without more information (Hendrickson et al., 2002; Lovley, 2003; Lu et al., 2006; Scheutz et al., 2008; Tas et al., 2010). Therefore, detecting genes involved in dehalogenation can better inform specific D. mccartyi catalytic capacity being expressed *in situ* in a groundwater system. For *D. mccartyi*, reductive dehalogenases (RDases) are a class of highly informative and field-site relevant gene targets. RDases are the enzymes that allow for respiration of chlorinated hydrocarbons, and multiple reductive dehalogenases (5–38 per strain) are encoded in the genomes of each strain of *D. mccartyi* (Löffler et al., 2013; Türkowsky et al., 2018). Various studies have shown that the expression of RDases is regulated, and only a subset of a genome’s full RDase repertoire is expressed at one time (Waller et al., 2005; Fung et al., 2007; Türkowsky et al., 2018). In addition to the RDases, many gene operons are present and syntenous across *D. mccartyi* strains (McMurdie et al., 2009). These operons include those that encode for proteins predicted to be directly involved in organohalide respiration (OHR) regardless of which halogenated organic molecule is serving as the electron acceptor. For example, hydrogen is the only electron donor used by *D. mccartyi* strains and the Ni-Fe hydrogen uptake hydrogenase, Hup, can serve as a potential biomarker of overall *D. mccartyi* strains OHR activity. These more generalized OHR biomarkers have been determined through several studies observing *D. mccartyi* Hup hydrogenase transcripts and proteins in different strains grown on various organohalide compounds (Morris et al., 2007; Marshall et al., 2012; Rowe et al., 2012; Heavner, 2013).

The quantitative correlation between reductive dechlorination activity and specific RNA transcripts has been previously explored (Johnson et al., 2005a,b; Waller et al., 2005; Rahm et al., 2006; Fung et al., 2007; Behrens et al., 2008; Lee et al., 2008; Rahm and Richardson, 2008a,b; Rowe et al., 2012; Schipp et al., 2013; Türkowsky et al., 2018). This quantification of *D. mccartyi* genes and transcripts was also previously extended to a monitoring campaign at a trichloroethene (TCE)-contaminated field site (Lee et al., 2008). Extending this approach, enzymes are expected to be superior indicators of activity compared to genes or RNA transcripts because the enzymes are the direct functional catalysts for activity. However, detecting these protein biomarkers is difficult, particularly in complex mixed communities such as those in soil aquifers. Thus far, no successful shotgun metaproteomic characterizations have been reported for biomass collected at a field site undergoing *in situ* bioremediation via *D. mccartyi*-containing mixed communities.

To extend knowledge covering DNA and RNA biomarkers to proteins, several studies have been conducted concerning the proteome of *D. mccartyi* (Morris et al., 2006, 2007; Adrian et al., 2007; Werner et al., 2009; Rowe et al., 2012; Tang et al., 2013; Schiffmann et al., 2014; Liang et al., 2015; Pöritz et al., 2015; Türkowsky et al., 2018). In addition to confirming abundant RDase expression, collectively these studies suggest that the HupL hydrogenase is the key hydrogenase for oxidation of hydrogen, the only electron donor for *Dehalococcoides.* Based on these studies, a preliminary suite of potential field biomarkers can be generated that includes RDases such as the vinyl chloride (VC) RDase (VcrA and BvcA) that have been linked to successful biological dechlorination of VC to ethene in groundwater systems (Scheutz et al., 2008). Because VC is the most toxic of the chlorinated ethenes [with an Environmental Protection Agency (EPA) maximum contaminant level (MCL) of 2 μg/L] and the biochemical transformation of VC to ethene is a critical terminal step during PCE/TCE bioremediation (Scheutz et al., 2008), proteins linked to the VC to ethene step of reductive dechlorination (VcrA, BvcA) may be the best biomarkers for monitoring the complete remediation of chlorinated ethenes (Cupples et al., 2003; Muller et al., 2004; Scheutz et al., 2008; Tang et al., 2013).

In this study, groundwater samples were collected from a well-characterized, TCE-contaminated industrial site in southern Ontario, Canada (ISSO) (Mundle et al., 2012; Pérez-de-Mora et al., 2014, Pérez de Mora et al., 2017). The presence of *cis*-DCE and VC in the site groundwater indicated that TCE-dechlorination by native microorganisms was occurring, albeit at slow rates. An enhanced *in situ* bioremediation (EISB) system was installed in 2008. Bioaugmentation with KB-1^TM^ was conducted in 2009, and in 2011 groundwater biomass was sampled and processed to extract DNA, RNA, and protein. The DNA and mRNA for seven selected *D. mccartyi* targets were quantified, with a focus on RDase genes and the uptake hydrogenase, *hupL*. Proteins of field samples were extracted, digested with trypsin, and analyzed by high resolution mass spectrometry. The field metaproteomes were compared with proteomes of the KB-1^TM^ seed culture, and relative protein abundances were compared to RNA transcript abundances to discover robust biomarkers for dehalogenation activity.

## Materials and Methods

### Site Description

Details of the site have previously been published (Mundle et al., 2012; Pérez-de-Mora et al., 2014, Pérez de Mora et al., 2017). The EISB system was installed about 3 years prior to sampling. The system extracts groundwater from the TCE-contaminated site through three wells located downgradient of the assumed source area (“EW” in Supplementary Figure S1) (Mundle et al., 2012). The extracted groundwater was amended with ethanol as an electron donor and re-injected into the aquifer through recharge wells (IW in Supplementary Figure S1) located in the vicinity of the source area, up-gradient of the extraction wells. After 1 year of operation, select recharge wells were bioaugmented with approximately 100 L of the commercially-available KB-1^TM^ microbial culture in October 2009 to enhance biotransformation rates of chloroethenes. In this study, four monitoring wells (Supplementary Figure S1) were sampled from the ISSO site – two with high hydraulic connectivity to the EISB zone (PM2A2 and EW1) and two with low hydraulic connectivity to the EISB zone (O-BH09-A1 and O-BH10-A1), with connectivity based on bromine tracer tests previously conducted by Geosyntec Consultants (Guelph, ON, Canada). All four samples originated from anaerobic locations (redox potentials between −250 and −300 mV) with documented TCE contamination and dechlorination activity (Supplementary Figure S2) (Pérez-de-Mora et al., 2014).

### Groundwater Sample Collection

Groundwater samples were collected approximately 2 years after bioaugmentation at the site. Samples were obtained from established wells using dedicated inertial lift pumps with traditional groundwater purging methods. Groundwater (2–4 well volumes) was purged from the well until water quality parameters [e.g., temperature, pH, dissolved oxygen (DO), redox potential, conductivity, and turbidity] had stabilized when measured using a flow-through cell attached to a multi-parameter sensor. Additionally, the water elevation was monitored before and after sampling. Between 8 and 12 L of groundwater were collected in collapsible, 5 gallon containers and transported to the North Treatment Building (NTB) on site for immediate filtering.

For each sample, 8–12 L of groundwater were filtered through a prefilter (1.2 μm pore size, 142 mm diameter, Versapor^®^Acrylic Copolymer Membrane Disc Filters; Pall Corporation, Port Washington, NY, United States) followed by a filter (0.2 μm pore size, 142 mm diameter, Supor^®^PES Membrane Disc Filters; Pall Corporation, Port Washington, NY, United States). Exact volumes filtered are presented in Supplementary Table S1. The groundwater was pumped at approximately 0.5 L/min. Each filter was then removed from the filtering apparatus, folded, placed in a sterile 50-mL conical tube, and flash frozen in an ethanol/dry ice bath before being stored on dry ice. A total of 10 6-mm diameter filter pieces (11 for well EW-1) were removed with a standard hole punch as subsamples for nucleic acid extraction, and the remaining filters were shipped overnight on dry ice to the Environmental Molecular Sciences Laboratory (EMSL) at the Pacific Northwest National Laboratory (PNNL) for proteomic analysis.

### Nucleic Acid Extraction and Quantification

DNA and RNA extractions were performed using the AllPrep DNA/RNA Mini Kit (the RNA was subsequently DNase treated; Qiagen) as previously described (Rahm et al., 2006; Rowe et al., 2012). In place of a cell pellet, 5 hole punches (1.3% of the filter surface area) were placed in the cell lysis tube for nucleic acid extraction. DNA and RNA extractions were performed within 24 h using UltraClean microbial DNA isolation (Mo Bio Laboratories) and RNeasy Mini (QIAGEN) kits. For RNA samples, reverse transcription inefficiencies and mRNA losses incurred during sample preparation were estimated using a modified normalization protocol in which 6 × 10^9^ copies of luciferase control RNA (Promega) were added to cell pellets prior to lysis (Johnson et al., 2005a). RNA was quantified using the RNA 6000 Nano assay on an Agilent 2100 bioanalyzer (Agilent Technologies). Total DNA was quantified using the Quant-iT^TM^ Picogreen^®^double stranded DNA assay (Invitrogen). RNA quality and quantity was analyzed using the Agilent 2100 BioAnalyzer. Secondary DNase treatment, cDNA synthesis, qPCR set up, and qPCR run conditions were performed as previously described (Rahm and Richardson, 2008a,b; Rowe et al., 2012). Prior to qPCR, all DNA and cDNA samples were diluted 1 to 5. Primers and annealing temperatures used in this study have previously been published (Rowe et al., 2012; Heavner et al., 2018). cDNA was synthesized from 0.1 μg of RNA with the iScript cDNA select synthesis kit using the provided random hexamer primers (Bio-Rad). Gene transcripts and added luciferase RNA were measured by amplification of corresponding cDNA using iQ SYBR Green Supermix (Bio-Rad) and DET-specific primers. Amplifications were performed in triplicate using an iCycler iQ multicolor real-time detection system (Bio-Rad) under conditions previously described (Rahm et al., 2006). Melt curve analysis confirmed the absence of primer-dimerization. Transcript levels were calculated from raw fluorescence data using the Data Analysis for Real-time PCR (DART-PCR) method (Peirson et al., 2003; Schefe et al., 2006; Rahm and Richardson, 2008a). Long amplicon or D2 mixed culture DNA extracts (for *hupL* and DET1559) were used to generate standard curves for each target to convert R_0_ values to copies (Rahm and Richardson, 2008a; Heavner et al., 2018).

### Sequencing of qPCR Products

qPCR products from amplification of DNA from the EW1 and O-BH9-A1 wells were purified using the QIAquick PCR Purification Kit (Qiagen). DNA sequencing was performed using an Applied Biosystems Automated 3730 DNA Analyzer at the Cornell University Life Sciences Core Laboratories Center. Trimmed sequences were analyzed using a NCBI- BLASTn query against the nr database.

### Proteomic Analysis

#### Proteomic Sample Preparation of Samples PM2A2 and EW1

Various tests were performed on filters PM2A2 and EW1 to determine the most appropriate filter extraction method. Filters were cut in half for two different procedures and subsequently cut into pieces for extraction. Urea (9 M) was added to the first half of the filter pieces, and the samples were vortexed, sonicated, and centrifuged. The cells in the supernatant were lysed via Pressure Cycling Technology (PCT) using a barocylcer (Pressure BioSciences, Inc., South Easton, MA, United States). The suspended cells were subjected to 20 s of high pressure at 35 kpsi followed by 10 s of ambient pressure for 10 cycles. The protein concentration was determined by a Coomassie assay (Thermo Scientific, Rockford, IL, United States) and reduced by adding 10 mM dithiothreitol (DTT). Samples were incubated at 60°C for 30 min, then diluted 10-fold with NH4HCO3 (100 mM, pH 8.4) to reduce the urea concentration. CaCl2 was added to the diluted sample to a final concentration of 1 mM, and the sample was digested for 3 h at 37°C using sequencing grade trypsin (USB, Santa Clara, CA, United States) at a ratio of 1 unit of trypsin per 50 units of protein (1 unit = ∼1 μg of protein). Following incubation, digested samples were desalted using an appropriately sized C-18 SPE column using Discovery C18 (50 mg, 1 mL) solid phase extraction tubes (Supelco, St. Louis, MO, United States) with the following protocol: 3 mL of methanol was added for conditioning followed by 2 mL of 0.1% TFA in H_2_O. The samples were then loaded onto each column followed by 4 mL of 95:5 H_2_O:ACN, 0.1% TFA, eluted with 1 mL 80:20 ACN:H_2_O, 0.1% TFA, and concentrated to ∼0.1 mL using a Speed Vac (Thermo Savant, Holbrook, NY, United States). The final peptide concentration was determined by bicinchoninic acid (BCA) assay (Thermo Scientific, Rockford, IL, United States).

Filter aided sample preparation (FASP) was used as an alternative sample preparation method (Wiśniewski et al., 2009; Jugder et al., 2016). The second half of the filter pieces had 2-mL Universal Protein Extraction (UPX) buffer (Expedeon, San Diego, CA, United States) added, and were sonicated, vortexed and barocycled as described above and incubated at 95°C for 5 min. Amicon Ultra-15 10K MWCO centrifuge devices (Millipore, Billerica, MA, United States) were used for buffer exchange. The sample was added to the filter unit with urea (8 M, pH 8.5) at 15% of the total volume. The unit was centrifuged at 4000 × *g* for 40 min (until it reached the dead volume of 200 uL). An additional 10 mL of urea (8 M, pH 8.0) was added. The unit was spun at 4000 × *g* for 40 min and this was repeated three times followed by three rinses with NH_4_HCO_3_ (100 mM, pH 8.0). A BCA assay was used to determine the protein concentration, and enough 25 mM NH_4_HCO_3_ was added to the filter unit to cover the filter. The flow-through collection tube was thoroughly cleaned and the sample was tryptically digested in a 1:50 (w/w) ratio with 1 mM CaCl_2_ and was allowed to incubate overnight at 37°C. The following day the peptide sample was centrifuged at 4000 × *g* for 30 min and the peptides were collected in the flow through. The filter was rinsed with NH_4_HCO_3_ (25 mM), and the flow-through was pooled. The sample was then cleaned via Strong Cation Exchange Solid Phase Extraction (SCX-SPE) (Supelco). Each column (100 mg, 1 mL) was conditioned with MeOH, and rinsed with ammonium formate in 25% ACN (10 mM, pH 3.0), ammonium formate in 25% ACN (500 mM), and nanopure water. Samples were acidified to a pH of 4.0 by adding 20% formic acid. Samples were then introduced to the columns and washed with ammonium formate in 25% ACN (10 mM, pH 3.0). Excess liquid was removed from the columns under vacuum. Peptides were eluted with 80:15:5 MeOH:H_2_O:NH_4_OH and concentrated to ∼100 μL using a SpeedVac. Final peptide concentrations were determined using a BCA protein assay.

Both extraction methods resulted in contamination of the MS column. Therefore, the peptides from both methods were combined and used for offline high pH RP C-18 fractionation to remove contamination (described below).

#### Proteomic Sample Preparation of Samples O-BH9-A1 and O-BH10-A1

Frozen filter pieces were crushed and added to a 50-mL falcon tube. Five mL of the UPX buffer was added to each and incubated at 40°C for 2 h to extract the sample. The solution was pipetted out and the proteins were precipitated with a methanol/chloroform extraction (MPLEx) to remove contaminants^1^. The protein pellet was dried lightly under N2. The sample was reduced and denatured with DTT/ urea, trypitically digested, and C18 SPE cleaned as described above. The peptides were then offline high pH RP C-18 fractionated.

#### High pH RP C-18 Fractionation

Peptide samples were diluted to a volume of 900 μL with an ammonium formate buffer (10 mM, pH 10.0) and resolved on a XBridge C18 (250 mm × 4.6 mm, 5 μM with 4.6 mm × 20 mm guard column, Waters, Milford, MA, United States). Separations were performed at 0.5 mL/min using an Agilent 1100 series HPLC system (Agilent Technologies, Santa Clara, CA, United States) with mobile phases (A) ammonium formate (10 mM, pH 10.0) and (B) 10:90 ammonium formate (10 mM, pH 10.0):acetonitrile. The gradient was adjusted from 100% A to 95% A over the first 10 min, 95% A to 65% A over minutes 10 to 70, 65% A to 30% A over minutes 70 to 85, maintained at 30% A over minutes 85 to 95, re-equilibrated with 100% A over minutes 95 to 105, and held at 100% A until minute 120. Fractions were collected every 1.25 min (96 fractions total). All fractions were dried to half their volume under vacuum. Every five fractions of the PM2A2 and EW1 sample fractions were pooled beginning at 40 min to ensure the contamination peaks were not collected. Every five fractions of the O-BH9-A1 and O-BH10-A1 sample fractions were pooled beginning at 20 min because of fewer contamination peaks. The fractions were then completely dried down and 15 μL of ammonium bicarbonate (25 mM) was added to each fraction for storage at −20°C until LC-MS/MS analysis.

#### Proteomic Sample Preparation of KB-1^TM^ Mixed Culture Sample

The metaproteome for KB-1^TM^ culture grown on TCE as electron acceptor and methanol and ethanol as electron donors was prepared and analyzed as described elsewhere (Heavner et al., 2018). Briefly, protein extractions were performed on a 50 mL culture cell pellet (14,000 × *g*, 10 min). The culture was pelleted, decanted, and then frozen at −20°C. The frozen pellet was shipped overnight on dry ice to EMSL at PNNL for proteome extraction and analysis. The cell pellet was resuspended in 9 M urea, vortexed and barocycled. The sample was then chemically reduced using DTT and diluted 10-fold for preparation for trypsin digestion. The resulting peptides were cleaned using Discovery C18 solid phase extraction tubes and then loaded onto a column followed by 4 mL of 95:5 H_2_O:ACN, 0.1% TFA. The sample was then eluted with 1 mL 80:20 ACN:H_2_O. The sample was concentrated down to ∼30 μL using a Speed Vac and a final assay was performed to determine the peptide concentration. An equal mass of each sample was aliquoted into fresh centrifuge tubes. For the 2D-LC system the columns used consisted of a first dimension SCX column and second dimension reversed-phase column. MS analysis was performed using a LTQ Orbitrap Velos ETD mass spectrometer (Thermo Scientific, San Jose, CA, United States) fitted with a custom electrospray ionization (ESI) interface. Orbitrap spectra (AGC 1 × 10^6^) were collected from 400 to 2000 m/z at a resolution of 60k followed by data dependent ion trap CID MS/MS (collision energy 35%, AGC 3 × 10^4^) of the 10 most abundant ions. A dynamic exclusion time of 60 s was used to discriminate against previously analyzed ions.

#### Data Analysis

MS/MS data was searched using SEQUEST against a peptide database constructed from a series of isolate genomes and metagenomic datasets and other known RDase sequences, using relatively conservative filters [cross correlation (Xcorr) values of 1.9 (+1), 2.2 (+2), and 3.5 (+3)] (Washburn et al., 2001). SEQUEST searched a protein database with no-enzyme rules, and no static or dynamic modifications. The parent ion tolerance was ±3 Da while the fragment ion tolerance was ±0.5 Da. The search allowed for up to four missed cleavages. Resulting peptide identifications were filtered using an MS-GF (Kim et al., 2008) cutoff value of SpecProb less than 1E-10 (Kim et al., 2008), leading to a q-value of less than 0.01 for 75% of the results at the spectrum level (and < 0.05 for 90% of the results). Q-value estimates were computed using subsequent MS-GF+ searches using a partially tryptic search of the decoy version of the original database, again without any static or dynamic modifications. The following genomic data was used in the searches for spectra matches: two metagenomes [KB1-UT (Hug et al., 2011) (IMG-M taxon ID: 2013843002) and the D2 mixed culture (Hug et al., 2011) which contains *D. mccartyi* strain 195 (IMG-M taxon IDs: 3300005370 and 2032320001)], *D. mccartyi* strains CBDB1 and 195; additional reductive dehalogenases from *D. mccartyi* strains BAV1, GT and VS; and other documented non-dechlorinating community members (*Geobacter lovleyi*; *Methanoregula boonei*; *Methanosaeta thermophila*; *Methanospirillum hungatei*), *Spirochaeta thermophila*, *Sporomusa* str. KB1, *Syntrophomonas wolfei*, and *Syntrophus aciditrophicus*). Resulting peptide identifications were filtered using an MS-GF (Kim et al., 2008) cutoff value to 1 × 10^−10^ (Kim et al., 2008). Relative protein quantities of biomarkers in shotgun proteomic samples were estimated by calculating the normalized spectral abundance factor (NSAF) (Zybailov et al., 2006). This technique accounts for biases in peptide abundances arising from protein length differences and from slight variability in sample loading onto the LC instrument (Wilkins et al., 2009).

## Results and Discussion

### DNA and RNA Biomarker Trends

**FIGURE 1:**
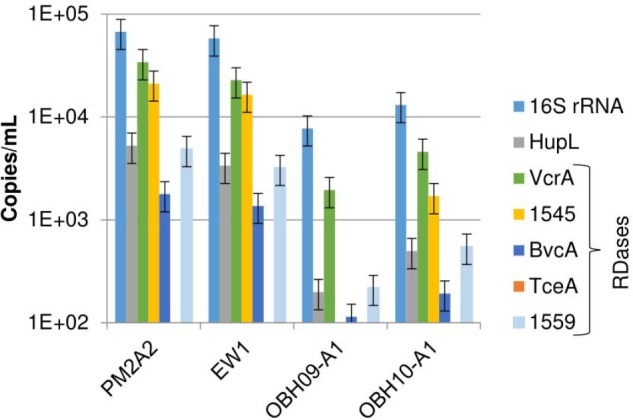
DNA biomarkers detected in filtered groundwater collected from monitoring well PM2A2, extraction well EW1, and background wells O-BH9-A1 and O-BH10-A1. The data in this figure are from DNA extracted off the filters (as opposed to the pre-filters). The detection limit is approximately 100 copies/mL. tceA was below detection in all samples. Error bars represent average standard deviations of replicate samples (*n* = 3–6).

The study site contained sampling wells from inside the active bioremediation zone as well as control wells outside the active zone (Supplementary Figure S1). Reductive dechlorination of chloroethenes was observed in wells in the active zone (PM2A2 and EW1) starting with electron donor (ethanol) addition in July 2008, 3 years prior to sampling (Supplementary Figures S2A,B). After bioaugmentation in October 2009, the rate of reductive dechlorination rapidly increased, and by July 2010, almost all chloroethenes had been transformed (Supplementary Figures S2A,B). The site is known to have a native *D. mccartyi* population, prompting the attempt to biostimulate prior to bioaugmentation (Zila, 2011; Pérez-de-Mora et al., 2014). Chloroethene reductive dechlorination was observed over this time period in background wells outside the active *in situ* bioremediation zone (O-BH09-A1 and O-BH10-A1) although with no measured accumulation of the end product of complete dechlorination, ethene (Supplementary Figures S2C,D). As shown in Figure 1, the most prevalent DNA biomarkers identified via quantitative PCR (qPCR) of groundwater biomass from all wells were genes for the 16S rRNA gene of *D. mccartyi* (1E4 – 1E5 copies/mL of site water), followed by *vcrA* and homologs of the RDase from *D. mccartyi* strain 195, DET1545 (1E3 – 1E4 copies/mL). *bvcA*, *hupL*, and RDase DET1559 gene homologs were detected, but at lower levels (1E2 – 1E3/mL). The TCE-RDase gene, *tceA*, was not detected above a detection limit of 100 copies per mL of filtered groundwater. The 16S rRNA gene and RDase gene abundances at another bioaugmented, TCE-contaminated field site displayed similar results – the 16S rRNA, *vcrA*, and DET1545 homolog genes were the most abundant, with *bvcA* and *tceA* detected at lower levels (Lee et al., 2008). Varying levels of DNA biomarkers result from the presence of multiple strains of *D. mccartyi* at the ISSO site – including native populations and multiple strains of *D. mccartyi* in the KB-1^TM^ inoculum (Duhamel and Edwards, 2007; Wei et al., 2016; Pérez de Mora et al., 2017). Although *hupL* is present in all strains of *D. mccartyi*, the *hupL* primers initially used in this study were specific for *D. mccartyi* strain 195 which may explain the low ratio of gene copies of *hupL* versus 16S rRNA genes (Figure 1). However, performing an additional qPCR with degenerate primers did not change that discrepancy (data not shown). The RDase biomarkers examined are not present in all strains, although the DET1545-homolog is widely distributed in genomes sequenced to date. The RDase DET1545 primers used in this study targeted a wide range of *D. mccartyi* strains encoding the DET1545 homolog (e.g., *D. mccartyi* strain 195, VS, CBDB1, GT, FL2 and BAV1, though in BAV1 the gene is interrupted and presumed non-functional) and the KB-1^TM^ mixed culture. However, they do not hit any RDase from strain MB (which produces *trans*-DCE rather than *cis*-DCE and has a truncated version of DET1545) (Cheng and He, 2009). BLAST queries of the sequenced qPCR products confirmed amplicons of *vcrA* and DET1545 but could not confirm *hupL* amplicons because of the short amplicon length (<100 base pairs).

**FIGURE 2:**
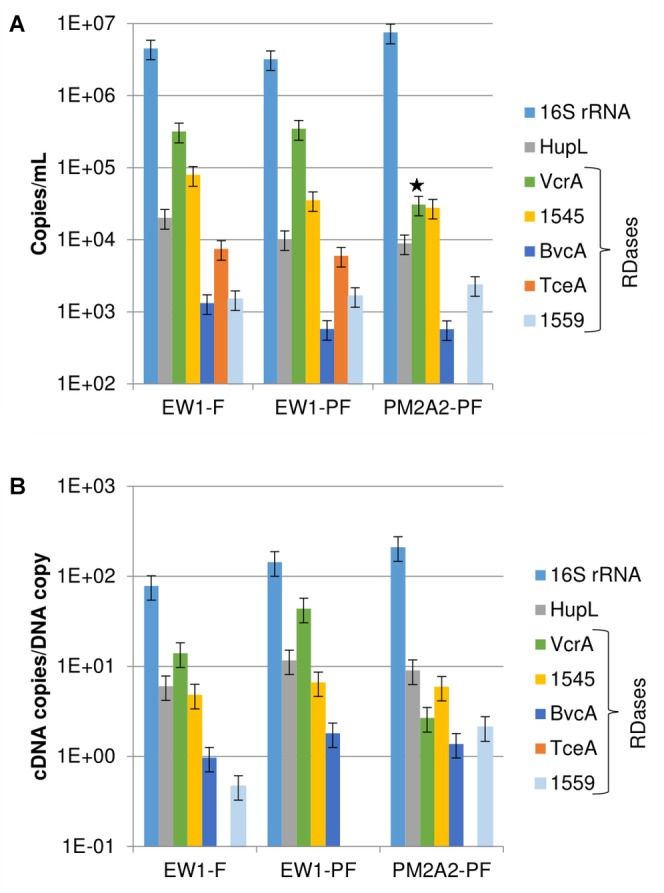
Transcript abundances. **(A)** cDNA copies per mL of groundwater. **(B)** cDNA copies per DNA copy for detected biomarkers in the PM2A2 and EW1 groundwater samples on DNA recovered from filters (F) and prefilters (PF). Error bars represent average standard deviations of replicate samples. Note that the PM2A2 cDNA from the filter was lost prior to qPCR and prefilter data alone is presented. The star indicates that the PCR efficiency of the VcrA sample for PM2A2-PF was outside the accepted range of 0.8–1.2, and that the value may be erring low. Note that transcripts of tceA were observed in EW1 but gene copies were below the detection limit of approximately 100 copies/mL.

RNA was successfully recovered in samples from monitoring wells EW1 and PM2A2. The RNA extracted from wells O-BH9-A1 and O-BH10-A1 was below quantification limits and was not further analyzed. As shown in Figure 2A, the most abundant RNA biomarkers observed in ground water samples for the ISSO site were 16S rRNA (1E6/mL), *vcrA* (1E4-1E5/mL), the DET1545 homolog (1E4/mL), and *hupL* (1E4/mL). *bvcA*, *hupL*, *tceA*, and DET1559 homologs were detected, but at lower levels (range: 1E2–1E3/mL). These trends follow those observed for DNA. However, the range of observed concentrations of biomarkers was larger for cDNA than for DNA, suggesting differential transcription of the different targets. As shown in Figure 2B, cDNA copies per DNA copy ranged from approximately 200 for 16S rRNA down to 0.5 for the DET1559 homolog (*tceA* cDNA was detected, although the gene was below detection limits in DNA samples). These ratios are similar to those found at another bioaugmented, TCE-contaminated field site and actively growing lab mixed cultures (Lee et al., 2008; Rowe et al., 2012). Prefilters were used in this study only to protect the filter from clogging. However, a significant percentage of the *D. mccartyi* gene copies were retrieved from the prefilters. Strong correlations were observed between the filter and pre-filter samples for DNA from wells PM2A2 and EW1 (Supplementary Figure S3A) and cDNA from well EW1 (Supplementary Figure S3B) with *R*^2^ values of 0.95 and 0.98, respectively. The slope of these curves suggest that approximately one third of the *D. mccartyi* (cells ∼0.5 μm in diameter) are being retained on the 1.2 μm pore size pre-filters, suggesting many of the cells are part of biofloc assemblages.

### Proteomic Results

#### Comparison of Field Proteome to Lab Proteome

**FIGURE 3:**
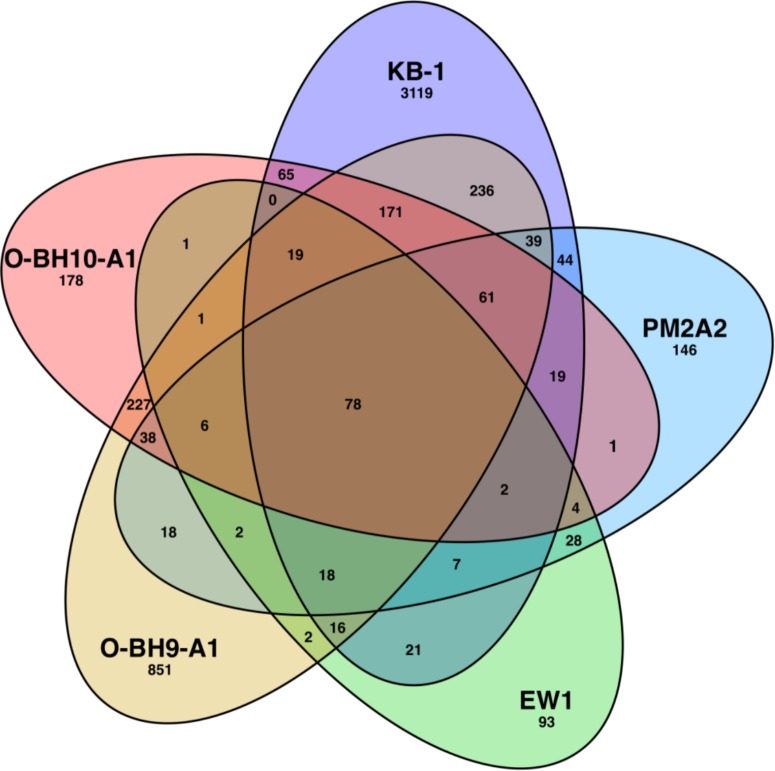
Overlap of detected proteins in the KB-1^TM^ mixed culture and the PM2A2, EW1, O-BH9-A1, and O-BH10-A1 field samples. Numbers indicate the number proteins matching detected peptides. The searched databases include multiple phylogenies beyond *Dehalococcoides*, including methanogens, fermenters, *Geobacter* and metagenomes for OHR enrichment cultures KB-1 and D2. This figure was generated from a template devised by Branko Gruenbaum and rendered by CMG Lee that is licensed under the Creative Commons Attribution-Share Alike 3.0 Unported license.

Using a concatenated gene library of open reading frames from various OHRB genomes, OHRB community metagenomes, and genomes of fermentative and methanogenic pure cultures, we detected peptide mass spectra from a total of 507, 298, 1779, and 871 proteins in the PM2A2, EW1, O-BH9-A1, and O-BH10-A1 samples, respectively, as compared to 3911 in the KB-1^TM^ mixed culture sample. These lower numbers likely result from the complexity (both phylogenetic and geochemical) of the field samples and an insufficient metagenomic database for the resident soil microbial community. Higher total numbers of proteins were detected in the O-BH9-A1 and O-BH10-A1 samples as compared to the PM2A2 and EW1 samples, despite an order of magnitude lower total populations of *D. mccartyi* (∼1 × 10^4^ *D. mccartyi*/mL versus 1 × 10^5^ *D. mccartyi*/mL). This may be because of issues originally encountered with the PM2A2 and EW1 samples as a result of precipitated minerals in these samples which required discarding of the first 40 min of nanoLC spectra for those samples. The lower *D. mccartyi* protein count in the EISB zone wells, PM2A2 and EW1, may also result from the relative population density of *D. mccartyi* strains because other phylogenetic groups besides OHRB are stimulated during EISB. In total, 78 proteins (across 18 unique homologous protein groups) were detected in all five samples (Figure 3 and Supplementary Table S2). These include VcrA and the DET1545 homolog from *D. mccartyi*, and several abundant proteins [e.g., ATP synthases, chaperonins, and methyl-accepting chemotaxis proteins] from various phylogenetic groups including *Dehalococcoides*, *Geobacter*, Bacteroidetes, syntrophic fermenters (e.g., *Syntrophomonas*) and sulfate reducing bacteria (e.g., *Desulfovibrio*) (Supplementary Table S2).

#### Biomarker Comparison

As with the DNA results, the most prevalent proteomic biomarkers detected in the field samples were VcrA and the DET1545 homolog for all samples except for O-BH9-A1, in which the PceA homolog from *Geobacter lovleyi* was the highest detected OHR biomarker (Table 1). Supplementary Figure S4 graphically presents how those 15 OHR biomarkers (RDases and HupL) from Table 1 were distributed among the samples. Nine RDases were found only in the KB-1^TM^ metaproteome sample, and only two (VcrA and the DET1545 homolog) were found in all the samples. The VcrA and DET1545 homologs were detected in the samples collected within the EISB. However, in wells O-BH9-A1 and O-BH10-A1, a greater number of RDases were detected, and their normalized spectral abundance factor (NSAF) scores were more evenly distributed (Table 1). The Ni-Fe hydrogenase, HupL, was detected in all samples apart from PM2A2. BvcA was among the top 5 detected proteins in the KB-1^TM^, O-BH9-A1 and O-BH10-A1 samples with NSAF scores of 923 x1E-5, 105 x1E-5, and 68 x1E-5, respectively, but was not detected in PM2A2 or EW1. This, combined with the cDNA/DNA levels (Figure 2) suggests that the *bvcA*-containing strain is expressing the *bvcA* gene at low levels. It is well-established that multiple strains of *D. mccartyi* are present in the KB-1^TM^ culture (Hug et al., 2012; Liang et al., 2015; Wei et al., 2016). The *Geobacter lovleyi* PceA homolog and the KB1_1 RDase (of unknown function) were detected only in the KB-1^TM^ mixed culture and sample O-BH9-A1.

**Table 1:** Detected reductive dehalogenase and Ni-Fe hydrogenase protein biomarkers in field samples compared to the KB-1^TM^ metaproteome sample.

			NSAF × 10^5^			
			Inside EISB		Outside EISB	
			
Homolog	OG#	KB-1^TM^	PM2A2	EW1	O-BH9-A1	O-BH10-A1
VcrA	8	4723	2825	2624	384	516
DET1545	15	4684	382	488	155	140
HupL	n.a.	959	<16	76	107	197
BvcA	28	923	<16	<31	105	68
KB1_8, KB1_9	17	66	<16	<31	<2.6	<7.1
DET0180	23	62	<16	<31	<2.6	<7.1
KB1_1	10	57	<16	<31	54	<7.1
TceA	5	49	<16	<31	<2.6	<7.1
KB1_1549	49	13	<16	<31	<2.6	<7.1
DET1528	75	8.4	<16	<31	<2.6	<7.1
KB1_7	19	7.7	<16	<31	<2.6	<7.1
DET1519	32	2.2	<16	<31	<2.6	<7.1
DET1538	71	1.1	<16	<31	<2.6	<7.1
cbdbA80	52	1.1	<16	<31	<2.6	<7.1
*G. lovelyi* PceA	n.a.	94	<16	<31	537	<7.1

*When more than one homolog was detected, the NSAF is a sum of NSAF’s for all detected homologs of an enzyme. The detection limit is based on the lowest NSAF for that sample (i.e., if a protein’s peptides were only found in one spectra in the whole spectral library). Shaded cells highlight where individual biomarkers were above the detection limit. OG#, Ortholog Groups of DMC reductive dehalogenases as defined by Hug et al. (2013) and Pérez de Mora et al. (2017). n.a., not applicable.*

Although the *Geobacter* PceA was not detected in all samples, other *Geobacter* proteins were detected in all samples (Supplementary Table S2). Most *Geobacter* species do not harbor *pceA* genes and rely on other types of respiration besides OHR (most notably metal reduction) (Türkowsky et al., 2018). Seven of the detected *Geobacter* proteins were detected in all samples but could be expressed by non-OHR species of *Geobacter* (Supplementary Table S2). Isocitrate dehydrogenase (Glov_1624) was detected in all four field samples, as well as the KB-1^TM^ sample, and citrate synthase (Glov_1379) was detected in three samples with the exception being O-BH10-A1. The detection of these central carbon metabolism proteins are important because they have previously been used to infer *Geobacter* metabolic activity in a field proteome study at a uranium-contaminated site in Rifle, CO, United States (Wilkins et al., 2009), and citrate synthase has been identified as a biomarker of *Geobacter* activity (Wilkins et al., 2011). Analyses of replicate and/or repeated samples would be needed to confirm if the metaproteome differences between background and EISB well are statistically significant.

#### Strain-Resolution Using Metaproteome Results

While the DET1545 homolog is present in many *D. mccartyi* strains (e.g., cbdbA1638/DhcVS_1436) and has previously been detected in DNA and RNA from field sites (Lee et al., 2008), *vcrA* and *bvcA* are less ubiquitous RDase genes. DET1545-homolog variation can be used to resolve strains in KB-1^TM^ (Heavner et al., 2018) and is informative for differentiating *D. mccartyi* strains because regions of strain variation were observed in the peptides detected for the KB-1^TM^ mixed culture. However the detected DET1545-homolog peptides did not allow for strain resolution (Cornell, VS, or Pinellas subgroups) because the PM2A2 peptide and one of the O-BH9-A1 peptides aligned with all three groups and the EW1, O-BH10-A1, and additional O-BH9-A1 peptides aligned with the Cornell and Pinellas subgroups (Supplementary Figure S5 and Supplementary Table S3).

**FIGURE 4:**
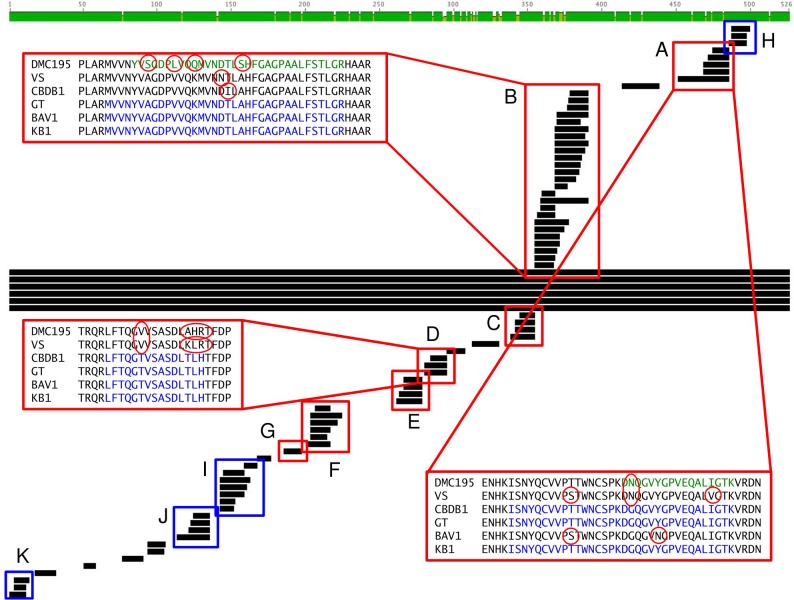
A comparison of HupL-homolog peptides detected in the KB-1^TM^ culture sample to the HupL-homolog sequences of *D. mccartyi* strain 195, *D. mccartyi* strains VS, CBDB1, GT, BAV1 and the dominant *D. mccartyi* strain in the KB-1 mixed culture. Peptides boxed in red **(A–G)** show strain specificity, whereas peptides boxed in blue **(H–K)** are highly conserved and align across all strains. Consensus peptides are highlighted in blue, and peptide variations that were detected in are highlighted in green, with the differences circled in red. The consensus sequence is the combined sequence of all detected peptides that align with each other and with a *D. mccartyi* strain. This figure was created using Geneious version 5.0.4 created by Biomatters.

With regards to strain variation, the HupL biomarker is also informative. Although, it is highly conserved among *D. mccartyi* strains (91% identity between *D. mccartyi* strain 195 and strain VS, 89% identity between *D. mccartyi* strain 195 and strains GT, BAV1, and CBDB1, as determined through a BLAST query), some of the detected peptides are unique to specific *D. mccartyi* subgroups. Of the 74 HupL peptides that were detected in the KB-1^TM^ culture sample, 14 peptides hit *D. mccartyi* strain 195 alone, 28 peptides align with all five strains, five peptides align with the Pinellas group (CBDB1, GT, and BAV1) but not *D. mccartyi* strain 195 and VS, and 13 peptides align with VS, CBDB1, GT, and BAV1 but not *D. mccartyi* strain 195 (Figure 4 and Supplementary Table S4). Consequently, *D. mccartyi* subgroups in KB-1^TM^ could be differentiated by virtue of differences in the peptides assigned to HupL homologs. Four HupL peptides were detected in each of the O-BH9-A1 and O-BH10-A1 field samples (Supplementary Figure S6). These peptides were also detected in the KB-1^TM^ mixed culture sample. One peptide aligned with all strains (Supplementary Table S4). The other three peptides, however, did allow for subgroup differentiation. The “DGQGVYGPVEQALIGTK” peptide (Figure 4 and Supplementary Table S4) aligned with the Pinellas group and not the Cornell or VS groups. The “YENTPYEVGPLAR” and “LAHELSAIYSGR” peptides aligned with the VS and Pinellas groups, but not the Cornell group. No peptides unique to the Cornell group (Figure 4 and Supplementary Table S4) were found in the site groundwater samples. Though *D. mccartyi* strain resolution was not a direct objective of this work, proteomics can be used to differentiate strains and monitor subgroup/strain population distribution in bioreactors and bioremediation communities within groundwater.

Both DNA analysis and proteomics provide complimentary evidence for the existence of different strains. Given all the possible sources of error in field sampling, obtaining additional lines of evidence from proteomics, that provide a more direct assessment of an active population of *Dehalococcoides* is valid. Clearly the status quo on biomarker monitoring is qPCR and RT-qPCR. PCR tools are much less expensive and methods can be very sensitive. However, they do suffer from “cross-talk” of biomarkers more so than MS-based peptide detection. If primers are near matches with non-target or near-target sequences, false positives must be controlled for carefully. Additionally, DNA does not tell you that the sub-population is necessarily active. We are not saying that you can’t use DNA for strain resolution, but that additional evidence from proteins detected in the field makes it easier to be convinced it is real. Currently, soil metaproteome monitoring presents many challenges for quantitative recovery of proteins from different cells as well as inherent variability of proteins with different hydrophobicity, size, tryptic peptide characteristics. Nonetheless, shotgun efforts such as the current study will naturally inform targeted peptide biomarker selection. Analogous to how metagenomes/metatranscriptomes lead to design of qPCR assays, metaproteomic studies can inform selection of more sensitive targeted approaches for lists of proteotypic peptides. As mass spectrometry tools become more routinely and used, it is likely that the value added of doing this may be warranted at times, depending on the questions and complexity of the site.

## Conclusion

This study provides the first report of proteomic data from EISB sites undergoing chloroethene bioremediation and extends to native organohalide respiring populations in groundwater microbial communities. Results revealed that *D. mccartyi* proteins can be detected in field samples, and that the detected RDases are a subset of those present in the KB-1^TM^ mixed culture that was used to bioaugment the site. Although the most highly detected OHR biomarkers were identical for DNA, RNA, and protein (VcrA and the DET1545 homolog), the benefits of using protein abundances over DNA and RNA include confirmation of complete gene expression and the potential for tracking microbial community shifts at the strain- or group-level. While strain differentiation is possible utilizing qPCR, the requirement of multiple primer sets for each target and issues associated with “cross-talk” make this a less viable option. Therefore, establishing and demonstrating a quantitative proteomic biomarker method could provide a more universal approach that directly quantifies the final enzymes, although issues with reproducible protein recovery remains a challenge for widespread diagnostic monitoring using analytical proteomics.

## Author Contributions

GH and CM conceived and designed the experiments and analyzed the data. GH, CM, MW, CN, and GD performed the experiments. EE provided access to the field site. RR contributed reagents, materials, and analysis tools. GH wrote the manuscript with assistance from CM, MW, CN, EE, and RR.

## Conflict of Interest Statement

While GH is currently affiliated with Floyd| Snider, Inc., the work described in the manuscript was carried out while GH worked at Cornell University. The remaining authors declare that the research was conducted in the absence of any commercial or financial relationships that could be construed as a potential conflict of interest.

## Footnotes

1 http://msystems.asm.org/content/1/3/e00043-16

## Supplementary Material

FIGURE S1 Schematic of the field site (ISSO). Groundwater is extracted from the northern portion of the property (EW wells) and transferred through buried piping to the northern treatment building (NTB) where it is combined, filtered, and amended with chlorine dioxide (ClO2) to control biofouling. The groundwater is then transferred to the southern treatment building (STB) through a central forcemain where the groundwater is amended with electron donor (ethanol) and distributed to individual recharge wells (IW). The wells sampled for this study were PM2-A2, EW1, O-BH9-A1, and O-BH10-A1.

FIGURE S2 Chlorinated ethene concentrations over time for monitoring well PM2-A2 **(A)**, extraction well EW1 **(B)**, background wells O-BH9-A1 **(C)**, and O-BH10-A1 **(D)** at an industrial site in southern Ontario, Canada. Electron donor addition began in July 2008 (solid vertical line) and bioaugmentation with KB-1^TM^ occurred in October 2009 (dashed vertical line). Samples were collected for nucleic acid and proteomic analysis in October 2011 (dotted line). Note that ethene was not analyzed in well O-BH10-A1.

FIGURE S3 Correlation of copies per mL on the filter (0.2 μm pore size) to the pre-filter (1.2 μm pore size) for DNA from EW1 and PM2A2 **(A)** and cDNA from EW1 **(B)** for detected biomarkers. cDNA from the PM2A2 from the filter was compromised and is not plotted. Points are colored by qPCR target. Error bars represent average standard deviations of replicate samples.

FIGURE S4 Overlap of detected *Dehalococcoide*s and *Geobacter* reductive dehalogenase, and HupL proteins in the KB-1^TM^ mixed culture sample and the PM2A2, EW1, O-BH9-A1, and O-BH10-A1 field samples. Numbers indicate the quantity of unique proteins detected.

FIGURE S5 Four regions of the DET1545 homolog where peptides were detected in the field samples (green) as compared to peptides detected in the KB-1^TM^ mixed culture sample (blue). Six homologs from *D. mccartyi* genomes and the KB-1 metagenome are shown in black. The amino acids that differ are circled in red if they match the *D. mccartyi* strain 195 sequence.

FIGURE S6 Four regions of the HupL-homolog where peptides were detected in the O-BH9-A1 and O-BH10-A1 field samples (green) as compared to the KB-1^TM^ mixed culture sample (blue). The amino acids that differ are circled in red if they match the *D. mccartyi* strain 195 sequence and in blue if they match the remaining sequences.

TABLE S1 Groundwater sample volumes and recoveries of DNA, RNA, and protein.

TABLE S2 Proteins detected in all five samples (KB-1 mixed culture and field samples PM2A2, EW1, O-BH9-A1, and O-BH10-A1). Raw Spectral counts and NSAF values provide for each metaproteome. Data sorted by highest to lowest NSAF score in KB1. Heat map with red showing high NSAF and green showing low NSAF scores.

TABLE S3 Consensus 1545 Homolog Peptides Detected in Shotgun KB-1TM Sample. Peptides are highlighted based on their specificity the Cornell group (green) or the Pinellas group (orange), and peptides highlighted in blue are highly conserved. See Figure 3 for peptide locations.

TABLE S4 Consensus HupL Homolog Peptides Detected in Shotgun KB-1TM Sample. Peptides are highlighted based on their specificity to the Cornell group (green) or the Pinellas group (orange), and peptides highlighted in blue are highly conserved.
