# Supplementary figures and images for: Detection of Organohalide-Respiring Enzyme Biomarkers at a Bioaugmented TCE-Contaminated Field Site
Source: Front Microbiol. 2019 Jun 27;10:1433. doi: 10.3389/fmicb.2019.01433 (PMC6610324; doi:10.3389/fmicb.2019.01433)

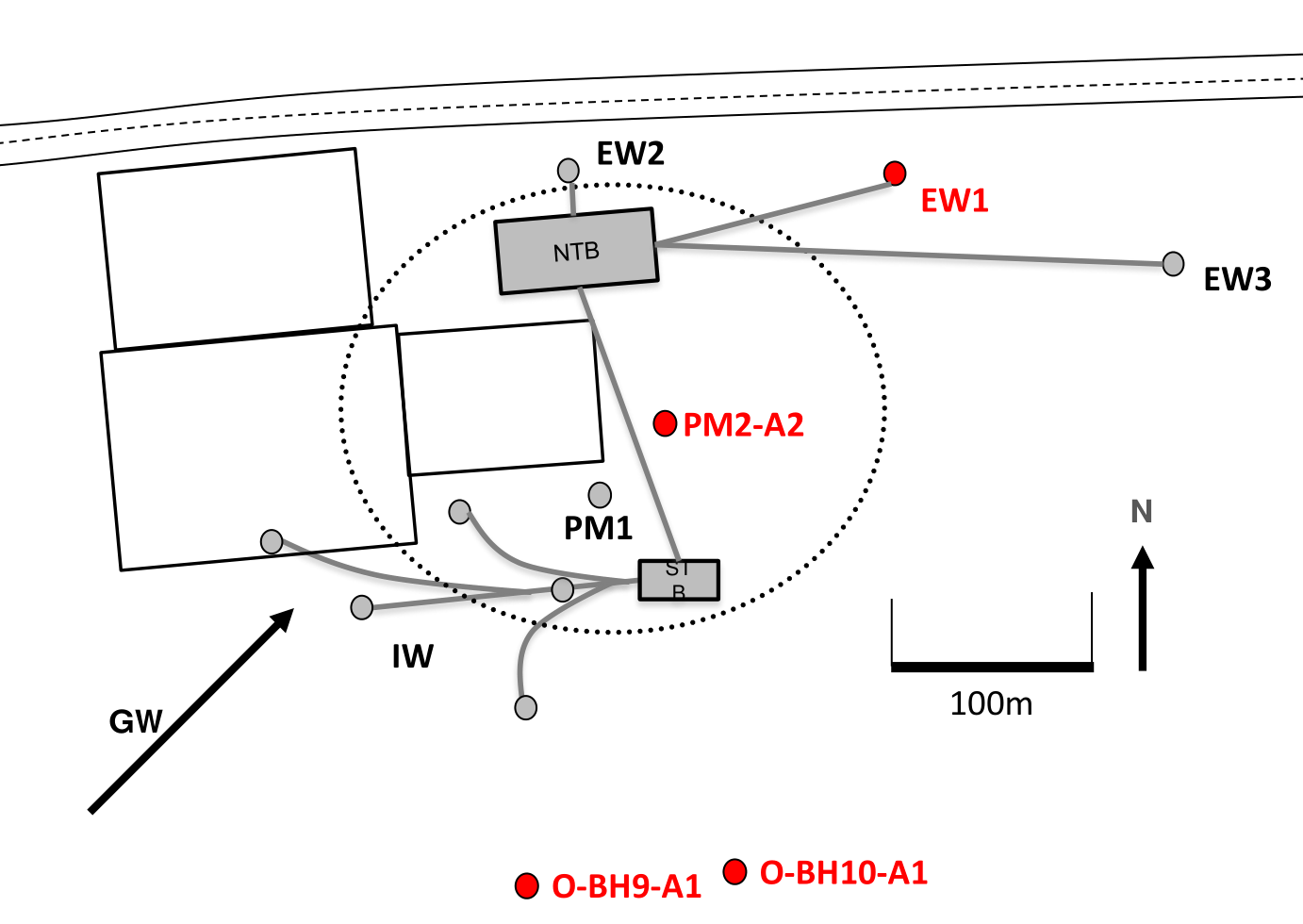

Supplement: FIGURE S1 — Schematic of the field site (ISSO). Groundwater is extracted from the northern portion of the property (EW wells) and transferred through buried piping to the northern treatment building (NTB) where it is combined, filtered, and amended with chlorine dioxide (ClO2) to control biofouling. The groundwater is then transferred to the southern treatment building (STB) through a central forcemain where the groundwater is amended with electron donor (ethanol) and distributed to individual recharge wells (IW). The wells sampled for this study were PM2-A2, EW1, O-BH9-A1, and O-BH10-A1. [file Image_1.TIFF]

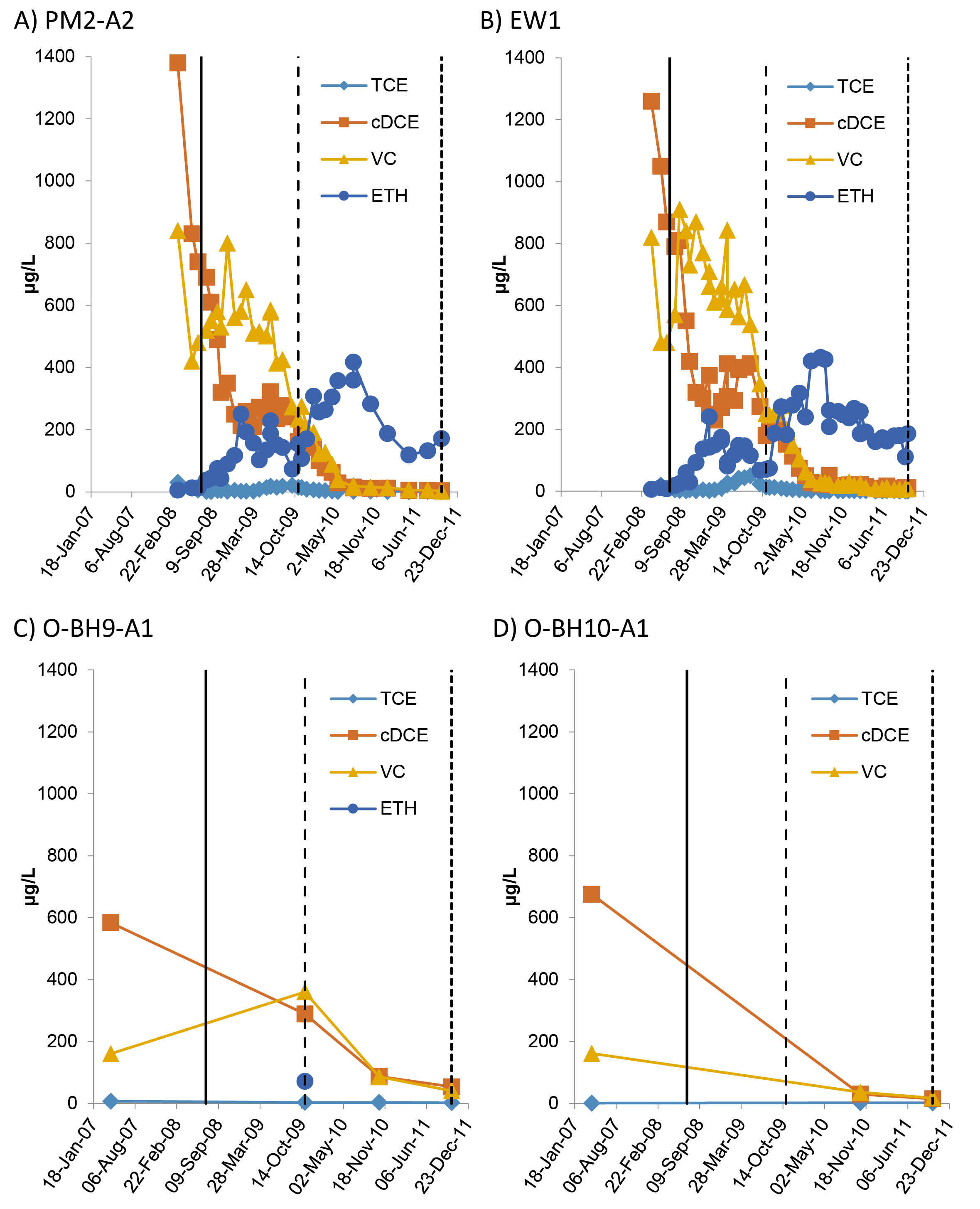

Supplement: FIGURE S2 — Chlorinated ethene concentrations over time for monitoring well PM2-A2 (A), extraction well EW1 (B), background wells O-BH9-A1 (C), and O-BH10-A1 (D) at an industrial site in southern Ontario, Canada. Electron donor addition began in July 2008 (solid vertical line) and bioaugmentation with KB-1TM occurred in October 2009 (dashed vertical line). Samples were collected for nucleic acid and proteomic analysis in October 2011 (dotted line). Note that ethene was not analyzed in well O-BH10-A1. [file Image_2.TIF]

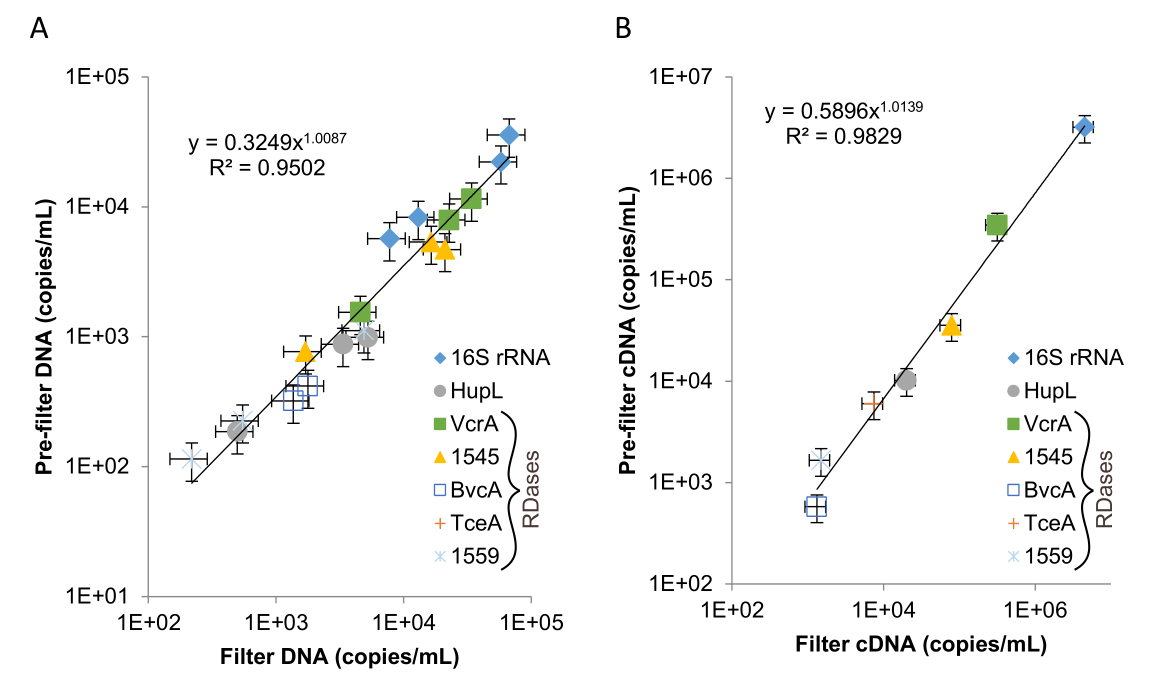

Supplement: FIGURE S3 — Correlation of copies per mL on the filter (0.2 μm pore size) to the pre-filter (1.2 μm pore size) for DNA from EW1 and PM2A2 (A) and cDNA from EW1 (B) for detected biomarkers. cDNA from the PM2A2 from the filter was compromised and is not plotted. Points are colored by qPCR target. Error bars represent average standard deviations of replicate samples. [file Image_3.TIFF]

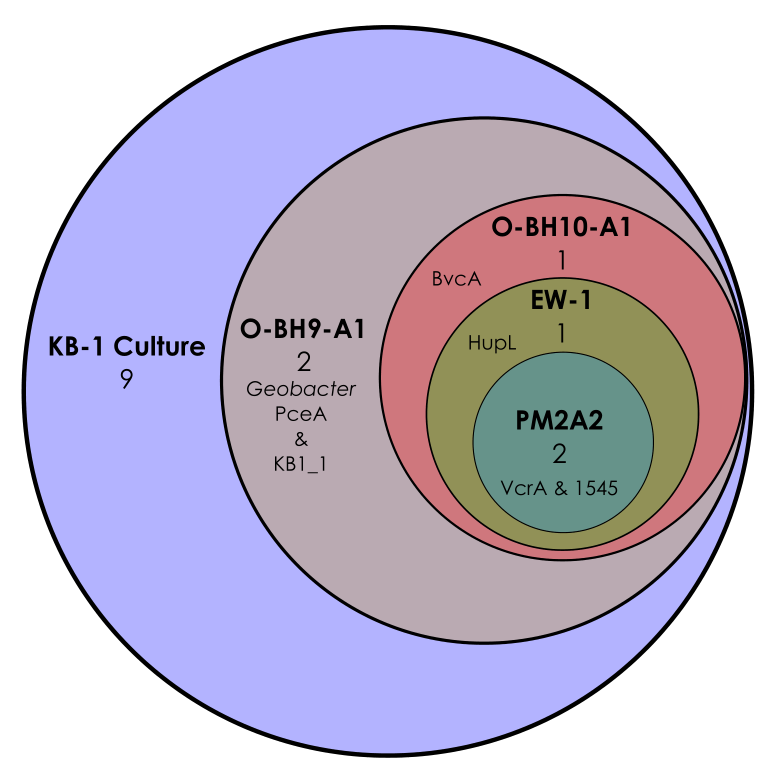

Supplement: FIGURE S4 — Overlap of detected Dehalococcoides and Geobacter reductive dehalogenase, and HupL proteins in the KB-1TM mixed culture sample and the PM2A2, EW1, O-BH9-A1, and O-BH10-A1 field samples. Numbers indicate the quantity of unique proteins detected. [file Image_4.TIFF]

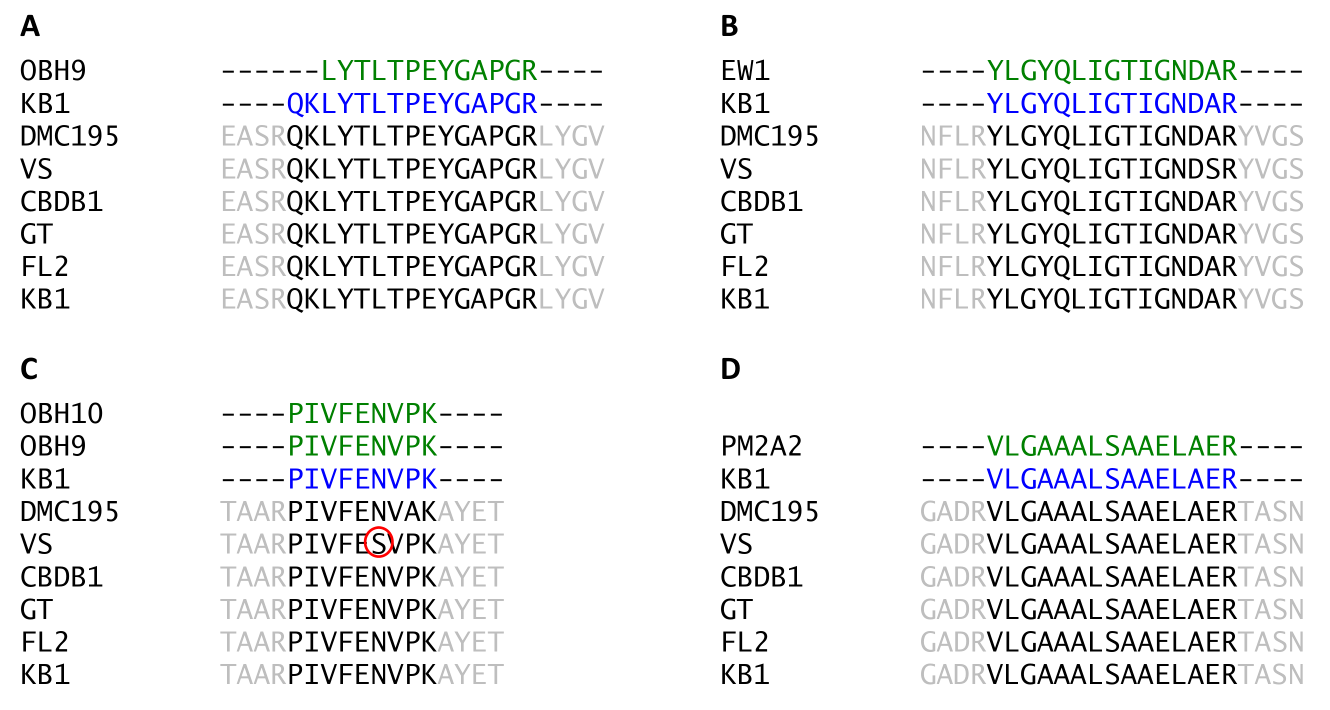

Supplement: FIGURE S5 — Four regions of the DET1545 homolog where peptides were detected in the field samples (green) as compared to peptides detected in the KB-1TM mixed culture sample (blue). Six homologs from D. mccartyi genomes and the KB-1 metagenome are shown in black. The amino acids that differ are circled in red if they match the D. mccartyi strain 195 sequence. [file Image_5.TIFF]

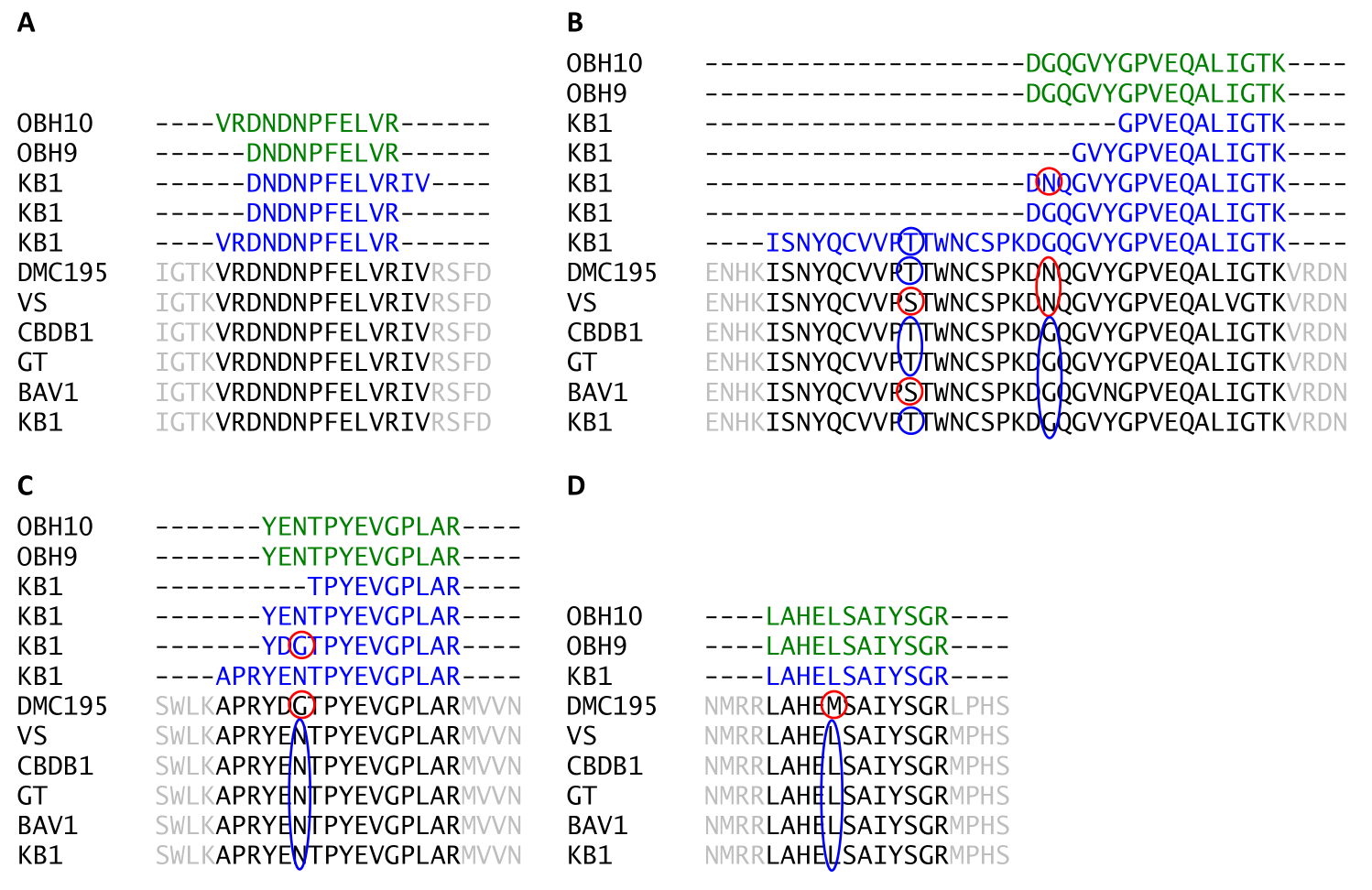

Supplement: FIGURE S6 — Four regions of the HupL-homolog where peptides were detected in the O-BH9-A1 and O-BH10-A1 field samples (green) as compared to the KB-1TM mixed culture sample (blue). The amino acids that differ are circled in red if they match the D. mccartyi strain 195 sequence and in blue if they match the remaining sequences. [file Image_6.TIFF]
