# Supplementary material for: Detection of Organohalide-Respiring Enzyme Biomarkers at a Bioaugmented TCE-Contaminated Field Site
Source: Front Microbiol. 2019 Jun 27;10:1433. doi: 10.3389/fmicb.2019.01433 (PMC6610324; doi:10.3389/fmicb.2019.01433)
Supplement: TABLE S1 — Groundwater sample volumes and recoveries of DNA, RNA, and protein. [file Data_Sheet_1.PDF]

Table S1. Groundwater sample volumes and recoveries of DNA, RNA and protein

Well	Volume Filtered (L)	Number of Hole Punches for Nucleic Acids Extraction	Volume Filtered Through Hole Punches (L)	Number of Hole Punches for Nucleic Acids Extraction	DNA recovered (ng)		RNA recovered (µg)		Protein recovered (µg)	
					F	PF	F	PF	F	PF
PM2A2	9.5	5	0.125	5	102	38	2.6	2.1	30	n/a
EW1	12	5	0.158	5	46	13	1.9	1.5	46	n/a
O-BH09-A1	11.25	5	0.148	5	2.8	2.5	bd	bd	60	n/a
O-BH10-A1	8.25	5	0.109	5	2.4	3.4	bd	bd	60	n/a

*bd means that RNA was below detection in extractant fluid.
n/a indicates not extracted.
