# Supplementary material for: Detection of Organohalide-Respiring Enzyme Biomarkers at a Bioaugmented TCE-Contaminated Field Site
Source: Front Microbiol. 2019 Jun 27;10:1433. doi: 10.3389/fmicb.2019.01433 (PMC6610324; doi:10.3389/fmicb.2019.01433)
Supplement: TABLE S2 — Proteins detected in all five samples (KB-1 mixed culture and field samples PM2A2, EW1, O-BH9-A1, and O-BH10-A1). Raw Spectral counts and NSAF values provide for each metaproteome. Data sorted by highest to lowest NSAF score in KB1. Heat map with red showing high NSAF and green showing low NSAF scores. [file Data_Sheet_2.PDF]

Table S2. Proteins detected in all five samples (KB-1 mixed culture and field samples PM2A2, EW1, O-BH9-A1 and O-BH10-A1). Raw Spectral counts and NSAF values provide for each metaproteome. Data sorted by highest to lowest NSAF score in KB1. Heat map with red showing high NSAF and green showing low NSAF scores.

				Spectral Counts					NSAF				
Protein #	Protein ID (NCBI or JGI/IMG-M)	Phylogenetic affiliation based on BLAST result	Description	KB1	PM2A2	EW1	O-BH9-A1	O-BH10-A1	KB1	PM2A2	EW1	O-BH9-A1	O-BH10-A1
1	gi|157326512	*Dehalococcoides*	reductive dehalogenase, VcrA	984	10	6	9	6	1.08E-02	4.67E-03	4.74E-03	9.04E-04	1.36E-03
2	DehalGT_1237	*Dehalococcoides*	reductive dehalogenase, VcrA	984	10	6	9	6	1.06E-02	4.58E-03	4.65E-03	8.86E-04	1.33E-03
3	DCKB1_96900	*Dehalococcoides*	reductive dehalogenase, VcrA	949	10	6	9	6	1.03E-02	4.58E-03	4.65E-03	8.86E-04	1.33E-03
4	gi|77176887	*Dehalococcoides*	reductive dehalogenase, VcrA	854	10	5	8	5	9.53E-03	4.72E-03	4.00E-03	8.13E-04	1.14E-03
5	DCKB1_10850	*Dehalococcoides*	hypothetical protein	1695	8	6	30	33	9.23E-03	1.85E-03	2.34E-03	1.49E-03	3.69E-03
6	gi|77176860	*Dehalococcoides*	reductive dehalogenase, DET1545 ortholog	553	1	1	2	1	6.41E-03	4.91E-04	8.31E-04	2.11E-04	2.38E-04
7	FL2_RdhA6	*Dehalococcoides*	reductive dehalogenase, DET1545 ortholog	551	1	1	2	1	6.39E-03	4.91E-04	8.31E-04	2.11E-04	2.38E-04
8	DCKB1_110110	*Dehalococcoides*	reductive dehalogenase, DET1545 ortholog	560	1	1	2	1	6.28E-03	4.75E-04	8.04E-04	2.04E-04	2.30E-04
9	cbdbA1638	*Dehalococcoides*	reductive dehalogenase, DET1545 ortholog	560	1	1	2	1	6.28E-03	4.75E-04	8.04E-04	2.04E-04	2.30E-04
10	DehalGT_1353	*Dehalococcoides*	reductive dehalogenase, DET1545 ortholog	560	1	1	2	1	6.28E-03	4.75E-04	8.04E-04	2.04E-04	2.30E-04
11	DCKB1_91570	*Geobacter*	translation elongation factor 1A (EF-1A/EF-Tu)(EC:3.6.5.3)	196	8	8	1	3	4.43E-03	7.67E-03	1.30E-02	2.06E-04	1.39E-03
12	DCKB1_11070	*Dehalococcoides*	chaperonin GroL	312	5	5	24	17	3.26E-03	2.21E-03	3.74E-03	2.28E-03	3.65E-03
13	Glov_1331	*Geobacter*	translation elongation factor Tu	213	7	8	3	6	3.02E-03	4.20E-03	8.12E-03	3.87E-04	1.74E-03
14	Glov_1344	*Geobacter*	translation elongation factor Tu	213	7	8	3	6	3.02E-03	4.20E-03	8.12E-03	3.87E-04	1.74E-03
15	DET1428	*Dehalococcoides*	co-chaperonin GroEL	231	5	5	20	12	2.41E-03	2.21E-03	3.74E-03	1.90E-03	2.57E-03
16	PCEOTH_831740	*Clostridiales*	ATP synthase F1 subcomplex beta subunit(EC:3.6.3.14)	40	1	3	6	2	2.14E-03	2.26E-03	1.15E-02	2.92E-03	2.19E-03
17	PCEOTH_2517890	*Actinobacteria*	ATP synthase F1 subcomplex beta subunit(EC:3.6.3.14)	34	1	3	6	2	2.01E-03	2.50E-03	1.27E-02	3.23E-03	2.42E-03
18	Glov_3170	*Geobacter*	ATP synthase F1, beta subunit (EC:3.6.3.14)	148	3	4	14	7	1.76E-03	1.51E-03	3.41E-03	1.52E-03	1.71E-03
19	DCKB1_131470	*Dehalococcoides*	degV family protein	82	1	1	20	12	1.65E-03	8.52E-04	1.44E-03	3.66E-03	4.95E-03
20	PCEOTH_619260	*Dehalococcoides*	ketol-acid reductoisomerase (EC 1.1.1.86)(EC:1.1.1.86)	28	2	1	1	1	1.37E-03	4.13E-03	3.50E-03	4.44E-04	1.00E-03
21	PCEOTH_1287470	*Bacteroides*	translation elongation factor 1A (EF-1A/EF-Tu)(EC:3.6.5.3)	33	11	7	5	1	1.31E-03	1.85E-02	2.00E-02	1.81E-03	8.17E-04

Protein #	Protein ID (NCBI or JGI/IMG-M)	Phylogenetic affiliation based on BLAST result	Description	Spectral Counts KB1	Spectral Counts PM2A2	Spectral Counts EW1	Spectral Counts O-BH9-A1	Spectral Counts O-BH10-A1	NSAF KB1	NSAF PM2A2	NSAF EW1	NSAF O-BH9-A1	NSAF O-BH10-A1
22	Glov_2019	*Geobacter*	Phosphopyruvate hydratase (EC:4.2.1.11)	94	3	1	3	3	1.22E-03	1.65E-03	9.33E-04	3.56E-04	8.01E-04
23	PCEOTH_2434430	*Bacteroides*	translation elongation factor 1A (EF-1A/EF-Tu)(EC:3.6.5.3)	30	11	7	5	1	1.17E-03	1.82E-02	1.95E-02	1.77E-03	8.00E-04
24	PCEOTH_726050	*Clostridiales*	ATP synthase F1 subcomplex beta subunit(EC:3.6.3.14)	28	1	3	6	2	1.16E-03	1.75E-03	8.87E-03	2.25E-03	1.69E-03
25	PCEOTH_3043890	*Clostridiales*	ATP synthase F1 subcomplex beta subunit(EC:3.6.3.14)	49	1	3	6	3	1.13E-03	9.74E-04	4.94E-03	1.26E-03	1.42E-03
26	DCKB1_106480	*Dehalococcoides*	ketol-acid reductoisomerase (EC 1.1.1.86)(EC:1.1.1.86)	64	2	1	3	2	1.08E-03	1.43E-03	1.21E-03	4.62E-04	6.94E-04
27	PCEOTH_878390	*delta-Proteobacteria*	ATP synthase F1 subcomplex beta subunit(EC:3.6.3.14)	24	1	3	7	2	9.97E-04	1.76E-03	8.93E-03	2.65E-03	1.71E-03
28	PCEDHC_06710	*Dehalococcoides*	BNR/Asp-box repeat./Fibronectin type III domain.	174	1	1	10	3	9.75E-04	2.37E-04	4.02E-04	5.11E-04	3.45E-04
29	DCKB1_21860	*Acetobacterium*	ATP synthase F1 subcomplex beta subunit (EC:3.6.3.14)	43	2	4	7	2	9.50E-04	1.87E-03	6.33E-03	1.41E-03	9.07E-04
30	PCEOTH_660870	*deltaProteobacteria*	ATP synthase F1 subcomplex beta subunit(EC:3.6.3.14)	23	1	3	10	2	9.35E-04	1.72E-03	8.74E-03	3.70E-03	1.67E-03
31	DET1407	*Dehalococcoides*	BNR/Asp-box repeat domain protein	165	1	1	10	3	9.25E-04	2.37E-04	4.02E-04	5.11E-04	3.45E-04
32	PCEOTH_240310	*actinobacteria*	ATP synthase F1 subcomplex beta subunit(EC:3.6.3.14)	27	2	3	6	2	8.66E-04	2.72E-03	6.89E-03	1.75E-03	1.32E-03
33	PCEOTH_145020	*Nitrospira*	ATP synthase F1 subcomplex beta subunit(EC:3.6.3.14)	19	1	1	1	1	7.61E-04	1.70E-03	2.87E-03	3.65E-04	8.22E-04
34	DET0831	*Dehalococcoides*	ketol-acid reductoisomerase(EC:1.1.1.86)	43	2	1	1	1	7.27E-04	1.43E-03	1.21E-03	1.54E-04	3.47E-04
35	PCEOTH_2733910	*clostridiales*	ATP synthase F1 subcomplex beta subunit(EC:3.6.3.14)	58	1	3	8	4	7.12E-04	5.20E-04	2.64E-03	8.95E-04	1.01E-03
36	PCEOTH_512590	*Geobacter*	Molecular chaperone	15	1	2	5	2	6.58E-04	1.86E-03	6.28E-03	2.00E-03	1.80E-03
37	DCKB1_41330	*Dehalococcoides*	ABC-type Fe3+-hydroxamate transport system, periplasmic component	41	1	1	7	1	6.57E-04	6.79E-04	1.15E-03	1.02E-03	3.29E-04
38	Glov_1624	*Geobacter*	isocitrate dehydrogenase, NADP-dependent (EC:1.1.1.42)	82	2	1	1	1	6.20E-04	6.41E-04	5.42E-04	6.89E-05	1.55E-04
39	DCKB1_138350	*distant to Chlorobi (85% aa ID)*	chaperonin GroL	58	3	3	8	6	5.97E-04	1.31E-03	2.21E-03	7.50E-04	1.27E-03
40	DCKB1_316130	*89% aa ID to Ignavibacteria*	ATP synthase F1 subcomplex beta subunit (EC:3.6.3.14)	28	3	4	6	4	5.78E-04	2.62E-03	5.91E-03	1.13E-03	1.69E-03
41	DCKB1_89510	*Geobacter*	ATP synthase F1 subcomplex alpha subunit (EC:3.6.3.14)	43	2	1	1	3	5.66E-04	1.12E-03	9.44E-04	1.20E-04	8.11E-04
42	DCKB1_356440	*weak ID to anything*	LSU ribosomal protein L12P	9	3	3	3	2	4.04E-04	5.70E-03	9.65E-03	1.23E-03	1.84E-03

Protein #	Protein ID (NCBI or JGI/IMG-M)	Phylogenetic affiliation based on BLAST result	Description	Spectral Counts KB1	PM2A2	EW1	O-BH9-A1	O-BH10-A1	NSAF KB1	PM2A2	EW1	O-BH9-A1	O-BH10-A1
43	PCEOTH_1239690	*Bacteroidetes*	Chaperonin GroEL (HSP60 family)	7	1	1	3	1	3.36E-04	2.03E-03	3.44E-03	1.31E-03	9.84E-04
44	PCEOTH_1796930	*Syntrophus*	Chaperonin GroEL (HSP60 family)	10	1	1	9	7	2.98E-04	1.26E-03	2.14E-03	2.45E-03	4.29E-03
45	Swol_2343	*Syntrophomonas*	ribosomal protein L7/L12	6	3	4	3	1	2.69E-04	5.70E-03	1.29E-02	1.23E-03	9.21E-04
46	PCEOTH_2546790	*distant to Aminobacterium*	LSU ribosomal protein L12P	6	3	3	2	3	2.67E-04	5.66E-03	9.57E-03	8.11E-04	2.74E-03
47	PCEOTH_682880	*Bacteroidetes*	Chaperonin GroEL (HSP60 family)	7	1	1	3	1	2.55E-04	1.54E-03	2.61E-03	9.95E-04	7.48E-04
48	Glov_2829	*Geobacter*	chaperone protein DnaK	28	1	2	6	3	2.46E-04	3.72E-04	1.26E-03	4.80E-04	5.41E-04
49	PCEOTH_2665550	*distant to Candidatus Aminicenantes bacterium RBG_13_62_12*	chaperonin GroL	23	4	1	7	9	2.39E-04	1.76E-03	7.46E-04	6.64E-04	1.92E-03
50	PCEOTH_2525800	*Cloacibacillus sp. An23]*	LSU ribosomal protein L12P	4	3	3	2	1	2.27E-04	7.20E-03	1.22E-02	1.03E-03	1.16E-03
51	PCEOTH_1752910	*Acetoanaerobium noterae*	LSU ribosomal protein L12P	4	3	3	3	2	1.84E-04	5.84E-03	9.89E-03	1.26E-03	1.89E-03
52	PCEOTH_1371030	*distant to Caloranaerobacter azorensis*	LSU ribosomal protein L12P	4	3	3	2	1	1.84E-04	5.84E-03	9.89E-03	8.38E-04	9.44E-04
53	DCKB1_40900	*Dehalococcoides*	DNA-directed RNA polymerase subunit beta' (EC 2.7.7.6)	31	1	1	2	3	1.34E-04	1.84E-04	3.10E-04	7.89E-05	2.67E-04
54	PCEOTH_821690	*Desulfovibrio*	Methyl-accepting chemotaxis protein	2	1	1	3	1	1.10E-04	2.33E-03	3.94E-03	1.50E-03	1.13E-03
55	PCEOTH_1250520	*Desulfovibrio aminophilus*	methyl-accepting chemotaxis sensory transducer	2	1	1	3	3	9.93E-05	2.10E-03	3.56E-03	1.36E-03	3.06E-03
56	PCEOTH_1120670	*distant to Syntrophorhabdus aromaticivorans*	Methyl-accepting chemotaxis protein	2	1	1	2	1	9.84E-05	2.08E-03	3.53E-03	8.97E-04	1.01E-03
57	PCEOTH_288530	*distant to Ornatilinea apprima*	Methyl-accepting chemotaxis protein	2	1	1	2	1	9.27E-05	1.96E-03	3.32E-03	8.45E-04	9.52E-04
58	PCEOTH_2999900	*deltaProteobacteria*	methyl-accepting chemotaxis sensory transducer	2	1	1	2	1	8.37E-05	1.77E-03	3.00E-03	7.63E-04	8.59E-04
59	PCEOTH_2283790	*Geobacter*	methyl-accepting chemotaxis sensory transducer	2	1	1	2	1	7.90E-05	1.67E-03	2.83E-03	7.20E-04	8.11E-04
60	PCEOTH_1104500	*distant to Nitrospira*	Methyl-accepting chemotaxis protein	2	1	1	2	1	7.63E-05	1.62E-03	2.73E-03	6.95E-04	7.83E-04

Protein #	Protein ID (NCBI or JGI/IMG-M)	Phylogenetic affiliation based on BLAST result	Description	Spectral Counts KB1	PM2A2	EW1	O-BH9-A1	O-BH10-A1	NSAF KB1	PM2A2	EW1	O-BH9-A1	O-BH10-A1
61	PCEOTH_2380250	*very distant to Nitrospira*	Methyl-accepting chemotaxis protein	2	1	1	2	1	7.29E-05	1.54E-03	2.61E-03	6.64E-04	7.48E-04
62	PCEOTH_1462300	*distant to Chloracidobacterium thermophilum]*	translation elongation factor 1A (EF-1A/EF-Tu)(EC:3.6.5.3)	1	4	2	10	4	7.19E-05	1.22E-02	1.03E-02	6.55E-03	5.90E-03
63	PCEOTH_1229430	*distant to Syntrophorhabdus aromaticivorans*	Methyl-accepting chemotaxis protein	2	1	1	3	1	7.19E-05	1.52E-03	2.58E-03	9.83E-04	7.38E-04
64	PCEOTH_866620	*distant to Dethiosulfovibrio salsuginis*	Methyl-accepting chemotaxis protein	2	1	1	3	1	7.06E-05	1.49E-03	2.53E-03	9.64E-04	7.24E-04
65	PCEOTH_2310650	*very distant to Sulfuricurvum sp. PC08-66*	Methyl-accepting chemotaxis protein	2	1	1	2	1	6.60E-05	1.40E-03	2.36E-03	6.01E-04	6.77E-04
66	PCEOTH_1721300	*distant to Leptolinea tardivitalis*	Methyl-accepting chemotaxis protein	2	1	1	2	1	6.17E-05	1.31E-03	2.21E-03	5.62E-04	6.33E-04
67	DCKB1_100500	*Geobacter*	methyl-accepting chemotaxis sensory transducer	2	1	1	2	1	5.94E-05	1.26E-03	2.13E-03	5.41E-04	6.09E-04
68	PCEOTH_2679690	*very distant to Eubacterium*	Methyl-accepting chemotaxis protein	2	1	1	3	1	5.78E-05	1.22E-03	2.07E-03	7.90E-04	5.94E-04
69	PCEOTH_411580	*distant to Leptolinea*	Methyl-accepting chemotaxis protein	2	1	1	3	1	5.15E-05	1.09E-03	1.84E-03	7.03E-04	5.28E-04
70	DCKB1_240970	*distant to Ignavibacteriales*	Methyl-accepting chemotaxis protein	2	1	1	3	1	5.05E-05	1.07E-03	1.81E-03	6.91E-04	5.19E-04
71	PCEOTH_323630	*distatnt to Leptolinea*	Methyl-accepting chemotaxis protein	2	1	1	3	1	4.86E-05	1.03E-03	1.74E-03	6.64E-04	4.98E-04
72	PCEOTH_904410	*deltaProteobacteria*	acetyl-coenzyme A synthetase (EC 6.2.1.1)(EC:6.2.1.1)	2	3	1	15	2	4.51E-05	2.86E-03	1.61E-03	3.08E-03	9.25E-04
73	DCKB1_185910	*Syntrophobacter*	ATP synthase F1 subcomplex alpha subunit (EC:3.6.3.14)	1	1	1	2	2	4.38E-05	1.86E-03	3.14E-03	7.98E-04	1.80E-03
74	PCEOTH_1323510	*distant to Syntrophomonas*	Methyl-accepting chemotaxis protein	2	1	1	2	1	3.94E-05	8.34E-04	1.41E-03	3.59E-04	4.04E-04
75	PCEOTH_335150	[illegible] *Anaerolinea*	[illegible] protein	2	1	1	3	1	3.03E-05	6.42E-04	1.09E-03	4.14E-04	3.11E-04
76	DCKB1_14180	*Dehalococcoides*	ABC-type oligopeptide transport system, periplasmic component	3	2	3	16	9	3.02E-05	8.52E-04	2.16E-03	1.47E-03	1.86E-03
77	Glov_3335	*Geobacter*	methyl-accepting chemotaxis sensory transducer	2	1	1	2	1	2.61E-05	5.53E-04	9.35E-04	2.38E-04	2.68E-04

Protein #	Protein ID (NCBI or JGI/IMG-M)	Phylogenetic affiliation based on BLAST result	Description	Spectral Counts					NSAF				
				KB1	PM2A2	EW1	O-BH9-A1	O-BH10-A1	KB1	PM2A2	EW1	O-BH9-A1	O-BH10-A1
78	DCKB1_174780	*Dehalococcoides*	Tfp pilus assembly protein, ATPase PilM	1	1	1	4	2	1.14E-05	4.81E-04	8.14E-04	4.14E-04	4.66E-04
