# Supplementary material for: Detection of Organohalide-Respiring Enzyme Biomarkers at a Bioaugmented TCE-Contaminated Field Site
Source: Front Microbiol. 2019 Jun 27;10:1433. doi: 10.3389/fmicb.2019.01433 (PMC6610324; doi:10.3389/fmicb.2019.01433)
Supplement: TABLE S3 — Consensus 1545 Homolog Peptides Detected in Shotgun KB-1TM Sample. Peptides are highlighted based on their specificity the Cornell group (green) or the Pinellas group (orange), and peptides highlighted in blue are highly conserved. See Figure 3 for peptide locations. [file Data_Sheet_3.PDF]

Table S3. Consensus 1545 Homolog Peptides Detected in Shotgun KB-1^{TM} Sample. Peptides are highlighted based on their specificity the Cornell group (green) or the Pinellas group (orange), and peptides highlighted in blue are highly conserved. See Figure 3.5 for peptide locations.

Peptide Numbers	Highlighted Region in Figure 3.5	Consensus Sequence	Combined Spectral Counts for Peptide	Cornell Group	Victoria Group	Pinellas Group and KB-1 Mixed Culture
13, 27, 26, 10, 9	A	GTIANIPLFNTYFYK	14			X
28	A	NVSLFNTYFYK	2	X		
14, 21, 20, 19, 37, 36, 45, 25, 24, 23, 22, 30	F	QKLYTLTPEYGAPGRLYGVLTDLPLEPTHPIDAGIYR	31	X	X	X
46, 12, 31, 44	B	YLGYQLIGTIGNDARYVGSEGGAAIMAGLGEASR	410	X	X*	X
32	C	SAGTLLGGMANGNTFYN	1			X
34, 15, 11, 18, 17, 16		LVIPNVPLWEIALSTQGSNELWR	8	X	X	X**
29, 43, 42	D	YIGTTIPVTAARPIVFENVPK	3			X
41		WTGTPEEASR	5	X		X
40		VSQGTSPGWAETK	2		X	X***
33, 35, 5, 4, 2, 1, 8, 39, 38	G	VLGAAALSAAELAERTASNYPGYTYR	81	X	X	X
3	E	AIYYGADR	1		X	X
7		ERPIDDPTIEVDF	1	X	X	X
6		DTAVQPRPWWVK	4	X	X	X

*3 of the detected peptides that form this consensus peptide don't hit VS, but 1 does

**5 of the detected peptides that form this consensus peptide don't hit FL2, but 1 does

***This peptide does not hit FL2
