# Supplementary material for: Detection of Organohalide-Respiring Enzyme Biomarkers at a Bioaugmented TCE-Contaminated Field Site
Source: Front Microbiol. 2019 Jun 27;10:1433. doi: 10.3389/fmicb.2019.01433 (PMC6610324; doi:10.3389/fmicb.2019.01433)
Supplement: TABLE S4 — Consensus HupL Homolog Peptides Detected in Shotgun KB-1TM Sample. Peptides are highlighted based on their specificity to the Cornell group (green) or the Pinellas group (orange), and peptides highlighted in blue are highly conserved. [file Data_Sheet_4.PDF]

Table S4. Consensus HupL Homolog Peptides Detected in Shotgun KB-1^{TM} Sample. Peptides are highlighted based on their specificity to the Cornell group (green) or the Pinellas group (orange), and peptides highlighted in blue are highly conserved.

Arbitrary Peptide ID Numbers	Consensus Sequence	Spectral Count	Cornell Group	Victoria Group	Pinellas Group			KB1-UT Mixed Culture
			DMC195	VS	CBDB1	GT	BAV1	
15,16,65	VRDNDNPFELVRIV	75	X	X	X	X	X	X
24,27	GVYGPVEQALIGTK	35	X		X	X	X*	X
12,30	ISNYQCVVPTTWNCSPKDGQGVYGPV EQALIGTK	11	X	X		X		
17	DNQGVYGPVEQALIGTK	2	X					
29	IGEPVIADYEIPETAEGMGLWEAPR	9	X					
45,46,51,52,53,74	MVVNYVSGDPLVQQMVNDTLSHFGA GPAALFSTLGR	28	X					
3,20,21	FGAGPAALFSTLGR	43	X	X	X	X	X	X
38,39,40,41,42,43,44, 62,63	MVNDTLAHFGAGPAALFSTLGR	180				X	X	X
47,48,49,50,61,64,73	MVVNYVAGDPVVQKMVN	31		X	X	X	X	X
10,59,72	APRYENTPYEVGPLAR	26		X	X	X	X	X
70	YDGTPYEVGPLAR	6	X					
22	GDTTEYPLNEVTEPEFTK	2	X					
58	TFDPSKITESIK	1	X					
26,36	LFTQGVVSASDLAHR	9	X					
35	LFTQGTVSASDLTLH	15			X	X	X	X
14	DLEASGTNLATR	1	X					
13,25,57	SYGVFDLEANGTNLATR	12		X	X	X	X	X
23,56,66,67,68,69	SVAVVAGGVTSHPSIDSISSFMSK	32			X	X	X	X
34	LAHELSAIYSGR	17		X	X	X	X	X
33	KVAQAAVAH	9	X	X	X	X	X	X
2,4,5,6,7,8,71	ALAAGDMSMLAPFYPRYEGDYRLPK	34	X	X	X	X	X	X
18,19,60	ALDYVDVTEVADYDGTDPELLK	4	X	X	X	X	X	X
9	ALDYVDVTEVADYDGTDPELLK	9	X		X	X	X	X
54,55	NLIQGANYIASH	4	X	X	X	X	X	X
1	AAFGVADKIPNNGR	2			X	X	X	X
11	DAVHITQR	5	X	X	X	X	X	X
28	IEATVDGGEVKDAK	1	X	X	X	X	X	X
31,32,37	MQKIVIDPITRIE	22	X	X	X	X	X	X

*One peptide doesn't hit BAV1
